# Self-Heating and Failure in Scalable Graphene Devices

**DOI:** 10.1038/srep26457

**Published:** 2016-06-09

**Authors:** Thomas E. Beechem, Ryan A. Shaffer, John Nogan, Taisuke Ohta, Allister B. Hamilton, Anthony E. McDonald, Stephen W. Howell

**Affiliations:** 1 Sandia National Laboratories, Albuquerque, New Mexico 87123, USA

## Abstract

Self-heating induced failure of graphene devices synthesized from both chemical vapor deposition (CVD) and epitaxial means is compared using a combination of infrared thermography and Raman imaging. Despite a larger thermal resistance, CVD devices dissipate >3x the amount of power before failure than their epitaxial counterparts. The discrepancy arises due to morphological irregularities implicit to the graphene synthesis method that induce localized heating. Morphology, rather than thermal resistance, therefore dictates power handling limits in graphene devices.

Beyond the curiosity of an atomic dimension, it is the transport properties of graphene that differentiates it from traditional materials. Exhibiting a mobility that can exceed 200,000 cm^2^/Vs and a thermal conductivity that can reach 2,000 W/mK, graphene is extremely efficient at transporting energy^1^,^2^. Because of this efficiency, graphene continues to be pursued for numerous microelectronic and optoelectronic applications^3^. Regardless of its final employment, common to each pursuit is the practical necessity of interfacing this two-dimensional material (2D) into a three-dimensional (3D) world at a scale practical for application. To this end, large-area, scalable, forms of graphene have been pursued since the materials first isolation leading to its now commonplace availability^4^,^5^. Graphene synthesized using chemical vapor deposition (CVD) atop copper, for instance, can be obtained at near wafer scale (*i.e.*, ~2 in.) from multiple vendors. Epitaxial (Epi) graphene realized *via* the sublimation of silicon (Si) from silicon carbide (SiC) is limited only by the size of the supporting substrate^6^.

The same syntheses processes that provide scale, however, also induce morphological imperfections that limit device performance. Both epitaxial and CVD syntheses result in small regions of non-uniform layer number^7^,^8^ that increase electrical resistance^9^. CVD devices require layer transfer that causes mobility degrading wrinkles and interlayer debris^10^,^11^. Similarly, strain can induce wrinkles limiting mobility for epitaxial devices^12^,^13^. Here, these morphological imperfections are shown to drive the self-heating and eventual failure of graphene devices.

Self-heating of exfoliated graphene devices has been examined previously using infrared-thermography (IR)^14^,^15^,^16^, Raman spectroscopy^17^,^18^, and scanning thermal microscopy (SThM)^18^. From these efforts, it has been shown that the heating of graphene evolves in a manner different than that of more traditional materials. First, heat generation does not take place uniformly across the active region of the device^14^,^15^. Defects such as wrinkles or grain boundaries, for instance, localize heating^19^. Additionally, variations in graphene’s carrier concentration that arise due to doping from the contacts or an external bias result in non-uniform electrical resistance and, by extension, heating. For this reason, temperature is not necessarily maximized in the middle of a graphene channel as would occur with uniform heat generation but instead where the electrical resistance is maximized^14^,^15^,^18^. Second, unlike traditional devices where the thermal conductivity of the active region chiefly dictates heat dissipation, it is the cross-plane coupling of heat from the graphene and into the substrate that is of primary consequence^17^,^20^. Thus, the thermal conductivity of the graphene itself is immaterial relative to the thermal properties of the materials upon which it rests.

Solely from the perspective of thermal resistance then, epitaxial graphene provides an avenue to minimize self-heating and maximize power dissipation. Silicon carbide has a thermal conductivity ~3x that of silicon at room temperature^21^,^22^. Furthermore, since epitaxial graphene is synthesized directly atop the SiC, there is no thermally insulative dielectric layer (*e.g.*, SiO_2_) separating the graphene from the more thermally conductive substrate. While transfer of CVD graphene to SiC can capitalize upon the high thermal conductivity of the substrate, weak van der Waals bonding will result in a comparatively large thermal resistance at the interface (*i.e.*, Kapitza resistance)^23^,^24^,^25^,^26^. Epitaxial growth, in contrast, possesses stronger bonding leading to a smaller thermal boundary resistance^27^.

Self-heating is not determined exclusively from the thermal resistance, however, but also the area over which heat is generated. Factors localizing heat generation will exacerbate its effect. Here, we demonstrate that morphological imperfections localizing heat generation in a graphene device ultimately determine its power handling capability. Specifically, the breakdown power of epitaxial graphene on SiC devices is shown to be <3x that of comparable devices made up of CVD graphene atop a similar substrate. Correlations between the temperature distribution obtained with infrared–(IR) thermography and the morphology acquired using Raman imaging indicate that the difference arises from heat localization stemming from morphological features characteristic of the synthesis process. Altogether, the results demonstrate that the morphology of the graphene, rather than the system’s thermal resistance, dictates the amount of power that it can sustain.

## Results

**Figure 1:**
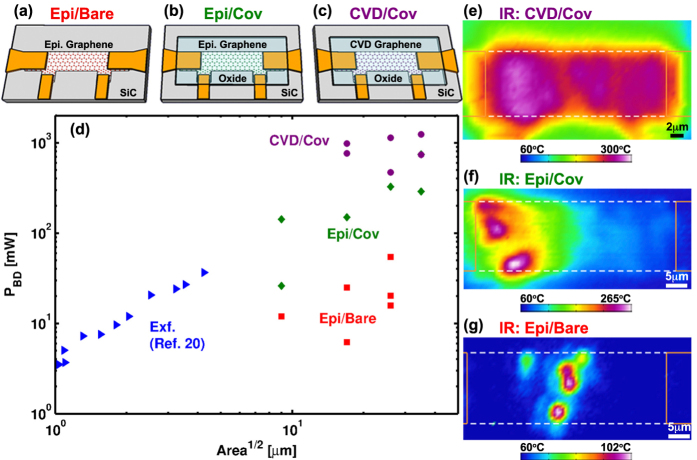
(**a–c**) Device architectures consisting of (**a**) epitaxial graphene exposed to atmosphere (Epi/Bare) (**b**) epitaxial graphene covered in an oxide (Epi/Cov) and (**c**) CVD graphene covered in an oxide (CVD/Cov). All devices rest on SiC substrates. (**d**) Breakdown power versus device area accompanied by (**e–g**) representative thermal images near failure for each of the device architectures. Higher breakdown powers correspond to more uniform heating. Reported temperatures are those directly measured by IR-thermography.

Three graphene device architectures were fabricated at chip scale using standard photo-lithographic processes as shown schematically in Fig. 1a–c. Two device architectures were built using quasi-free standing epitaxial monolayer graphene synthesized from (0001) 6H-SiC^28^,^29^. For one of the epitaxial devices, the graphene bar is left exposed to the atmosphere on its top side. This architecture is termed— epitaxial/bare (Epi/Bare). The other epitaxial architecture is coated with 50 nm of SiO_2_ deposited via plasma enhanced chemical vapor deposition. This is termed— epitaxial/covered (Epi/Cov). The final architecture is composed of commercially synthesized CVD graphene (ACS Materials) that is transferred to a SiC substrate and then covered with 50 nm of HfO_2_ realized *via* atomic layer deposition (ALD). This is termed—CVD/covered (CVD/Cov). Device geometries ranged in size from from 5 × 15 *μ*m to 20 × 60 *μ*m for each architecture and were contacted utilizing a Ti/Au stack (10/100 nm). Resistivity varied by ≤2x between the differing architectures and had comparable Raman responses largely absent of any defect signature (I(D)/I(G) ≤ 0.15, see Supporting Information).

For each architecture, six different devices of varying size were subject to increasing levels of Joule-heating until breakdown occurred. Breakdown was defined when channel resistance suddenly increased by several orders of magnitude. Only devices in which failure occurred in the channel—as opposed to the contacts—were included in the analysis. Practically, heating was induced by ramping current across the outer two leads to a target value held constant for 30s to facilitate thermography. Powers were measured by monitoring voltage across the inner contacts (see Fig. 1a–c). Utilizing a 4-wire arrangement provides a direct quantification of power dissipated within the graphene thereby allowing for comparisons between devices while minimizing the impact of variable contact resistance. For each power, the temperature distribution was imaged using a commercial IR-thermography system. Sufficient signal to noise in the IR signal was achieved by holding the bottom surface of the device at a temperature of 60 °C with a thermal stage. All measurements took place in ambient air and at steady state. Steady state was verified by ensuring stability of both the device’s I-V characteristics and that of the thermal map.

Breakdown power differs significantly between the architectures (see Fig. 1d). Quantitatively, the CVD/Cov devices fail at an average power >3x that of their epitaxial counterparts. Epitaxial devices exposed to the atmosphere (Epi/Bare), meanwhile, dissipate less than 10x that of their covered epitaxial complements (Epi/Cov) and nearly 40x less than covered devices consisting of CVD graphene (CVD/Cov). The variation in breakdown power is accompanied by qualitative differences in the nature of the heating. The CVD/Cov devices having the highest breakdown powers are observed to heat more uniformly (see Fig. 1e) than the less capable epitaxial based devices where heating localizes (see Fig. 1f,g).

Heating is of particular salience as the breakdown of graphene devices is thermal in origin^20^,^30^,^31^,^32^. Since the strength of the sp^2^ carbon bonds limits the susceptibility of the graphene to electromigration^33^, breakdown occurs instead when the graphene reaches a critical temperature sufficient to drive a reaction with the materials surrounding it^20^,^30^,^31^. Factors accelerating graphene heating—such as its localization—will therefore reduce power handling capability. With this paradigm, the large differences in power handling between device architectures are shown to emerge from morphological features that act to localize heat dissipation.

## Discussion

**Figure 2:**
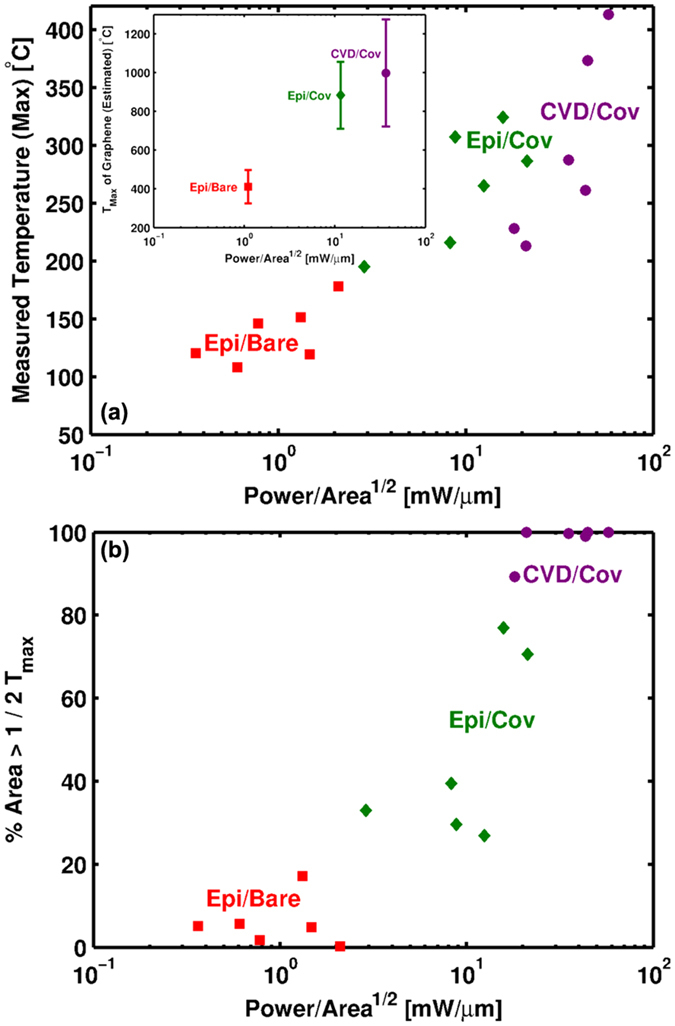
(**a**) Maximum temperature at failure as measured by IR-thermography. Inset: Maximum temperature of graphene at failure. Owing to volumetric averaging of the IR signal, the graphene temperature is ~3x larger than that measured by IR (see Supporting Information). (**b**) Percentage of device area possessing a temperature greater than 1/2 the maximum measured temperature. Devices capable of dissipating more power heat more uniformly.

Exposed to the environment, graphene reacts with oxygen upon reaching temperatures of ~450 °C^34^. Failure of a Joule-heated device will therefore initiate once the graphene reaches this critical temperature^20^,^32^. Upon initiation, oxidation will etch the graphene leading to breakdown of the conductive channel. Consistent with this interpretation, the bare epitaxial devices failed at an average graphene temperature of ~400 °C as shown by the inset of Fig. 2a. Graphene temperatures were deduced from the IR-maps by accounting for volumetric averaging using a procedure similar to Bae *et al*.^14^ (see Supporting Information). Thermoelastic effects are considered of secondary importance to the failure for two reasons. First, epitaxial graphene is subject to much larger temperature excursions during synthesis (Δ*T* > 1400 °C) than during the self-heating^13^. Second, failure of the architectures covered in an oxide occurred at similar temperatures for both epitaxial and CVD derived devices (see Fig. 2a). Since transferred CVD graphene has a much smaller residual stress than that of epitaxial^11^, its stress state will be much different during self-heating. Being different, thermoelastic driven failure would be expected to initiate at different temperatures if it were the prime causation. Failure is therefore concluded to be thermal, rather than themoelastic, in origin consistent with the discussion above.

Bare epitaxial devices failed at similar powers compared to previous reports of exfoliated devices (see Fig. 1d)^20^. This is counter to expectation for several reasons. First, exfoliated devices have a much larger substrate thermal resistance than epitaxial devices. Quantitatively, the SiO_2_/Si stack of an exfoliated device exhibits an out of plane thermal resistance of at least 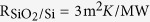 where the individual layer resistances are added in series and given by *R*_*i*_ = *L*_*i*_/*K*_*i*_ assuming thicknesses of 300 nm and 400 *μ*m for the SiO_2_ and Si, respectively. Thermal conductivities of the two layers are presumed to be: 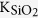 = 1.4 W/mK and *K*_*Si*_ = 148 W/mK^35^. In comparison, the thermal resistance of an epitaxial device atop 400 *μ*m of SiC (K_SiC_ = 450 W/mK) induces a resistance of R_SiC_ = 0.9 m^2^K/MW —3x less than the exfoliated condition. Second, the exfoliated device possesses two thermally resistive interfaces—graphene to SiO_2_ and SiO_2_ to Si—to the epitaxial devices one—graphene to SiC. Third, the exfoliated devices have an area that is at least 8x less than the epitaxial devices. Under the assumption of uniform heating, these effects together will cause the exfoliated devices to heat up much faster and, by extension, fail at significantly lower powers. This is contrary to observation as, in reality, the epitaxial devices do not heat uniformly.

**Figure 3:**
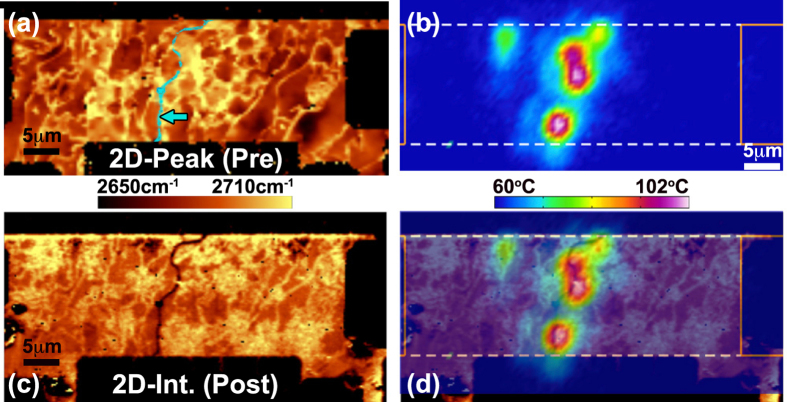
Raman and thermal imaging of a bare epitaxial graphene device. (**a**) Raman image of 2D-mode peak position before self-heating. Arrow highlights false color overlay of crack in (**c**). (**b**) Measured temperature distribution while dissipating 16 mW of power. This is not the graphene temperature but the volumetric average over the sampled volume of the IR measurement (See Supporting Information). (**c**) Raman image of 2D-mode intensity after failure where a crack is observed near the middle of the device. (**d**) Overlay of (**b,c**) showing that the crack is co-located with regions of most severe heating. The grounded electrode is on the right-hand side.

Figure 3 highlights the non-uniform heating of an epitaxial device acquired using IR-thermography where a series of localized hot-spots form across the device. These hot-spots remain stationary with increasing bias suggesting that they emerge not as a consequence of field induced carrier minimization but rather a cause independent to the electrostatics^15^. To probe this bias independent effect, Raman spectroscopic imaging was performed before and after device failure in order to compare graphene morphology to the emergence of these hot spots. From the Raman images, two observations are of consequence. First, no graphene signal is present after failure in the regions co-located with the hot-spots as evidenced by the “crack” seen in the 2D-mode intensity (see Fig. 3c,d). This was a consistent failure mode among the Epi/Bare devices and supports the conclusion of failure initiating upon reaching a critical temperature. Second, as seen in Fig. 3a, the hot spots form along boundaries exhibiting high variation in the 2D-peak position. The following examines the cause of this variance in the peak position and its implications on self-heating.

Epitaxial graphene emerges from the sublimation of Si from SiC resulting in concentration gradients of carbon that drive layer formation. The morphology of the underlying SiC affects the carbon concentration and thus the resulting graphene layer^8^. In practice, imperfections in the SiC morphology result in graphene that is non-uniform in layer number, carrier concentration^13^, and mobility^12^. These non-uniformities lead to localized regions of high electrical resistance that induce non-uniform heating, which ultimately limits power dissipation.

For example, graphene multilayers form near atomic steps of the SiC leading to monolayer multilayer boundaries^8^. In Fig. 3a, the multilayers are observed as thin “bright” regions running vertically across the device since the 2D-mode peak position shifts to higher wavenumber with increasing layer number^36^. At these junctions, the resistance increases significantly owing to the poor overlap between the wave-function of mono- and multilayer graphene^9^,^37^. Localized regions of high electrical resistance will lead to localized heating.

Similarly, electrical resistance, and by extension heating, will increase locally in regions of lower mobility. Lower mobility has been correlated with regions of high variation in the 2D-mode peak position^12^ that are present along single “terraces” away from the multilayer steps. Variations in 2D-mode peak position, in turn, evolve from the state of strain and carrier concentration in the epitaxial graphene that evolve due to interlayer interactions with the SiC substrate^13^. Significant variation in the 2D-mode peak position was observed near the region of failure indicating that these effects contribute to the localized heat generation as well (see Fig. 3a). Taken together, we therefore conclude that hot spots form in the epitaxial graphene owing to morphologically dictated regions of much higher electrical resistance that localize heating. Localized heating mitigates the advantages implied from the reduced thermal resistance of a SiC substrate. For this reason, the Epi/Bare devices fail at similar power levels as exfoliated devices having much larger thermal resistance and smaller sizes.

**Figure 4:**
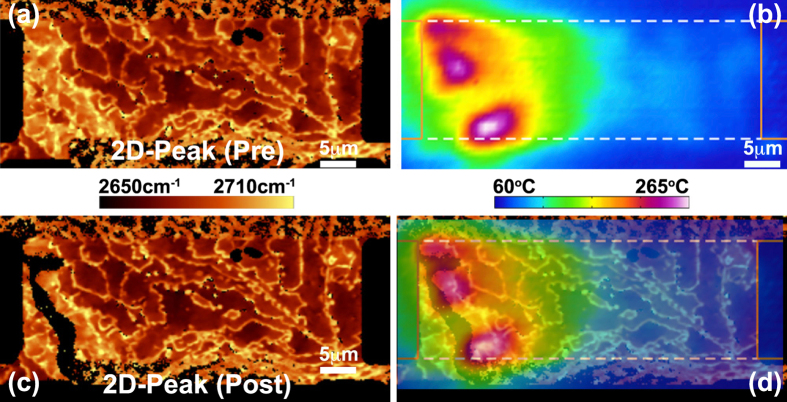
Raman and thermal imaging of a covered epitaxial graphene device. (**a**) Raman image of 2D-mode peak position before self-heating. Variation in peak position indicative of multilayer boundaries and mobility reductions are evident near left contact. (**b**) IR measured temperature distribution while dissipating 325 mW of power. (**c**) Raman image of 2D-mode peak position after failure. (**d**) Overlay of (**b,c**) indicating that the failure is co-located with regions of most severe heating. The grounded electrode is on the right-hand side.

Localized heating and failure was also observed in epitaxial devices covered in an oxide implying that hot spot formation observed in Fig. 3 is not a consequence of adsorbents or other environmental factors. Figure 4 provides Raman images of the 2D-peak position before and after failure along with the temperature field during heating. Near the left contact, a large concentration of multilayer steps are observed while on the terraces the peak position varies appreciably. As noted above, each of these morphological features will cause increased electrical resistance. Consequently, heating localizes in these regions leading to failure (see black “stripe” in Fig. 4c).

Relative to the Epi/Bare device, the Epi/Cov architecture is capable of withstanding much larger powers (see Fig. 1d). The higher powers are not indicative of any difference in graphene quality between the architectures or variations in their thermal resistance. Rather, the covered architecture dissipates more power because higher temperatures must be achieved before graphene begins to react with its surroundings—in this case the SiO_2_. SiO_2_ degradation initiates at temperatures of 900–1000 °C^38^,^39^,^40^,^41^. Upon reaching these temperatures, degradation allows mobile oxygen to come into contact with heated graphene thereby inducing failure^42^. In line with this interpretation, the graphene temperature at failure is comparable to the degradation temperature of SiO_2_ as shown by the inset of Fig. 2a.

Figure 2a also indicates that the temperature at failure measured by IR-thermography is similar for both the CVD/Cov and Epi/Cov architectures. This implies a common failure mode. Like SiO_2_, ALD layers of hafnia seeded with alumina begin to degrade near 900 °C^43^,^44^,^45^. As shown in the inset to Fig. 2a, the estimated temperature of graphene for the CVD/Cov architecture is comparable to this degradation temperature. It is therefore concluded that the CVD/Cov devices also fail upon the degradation of the dielectric covering graphene.

To summarize, the failure temperature of the CVD/Cov and Epi/Cov devices are each comparable to the degradation temperature of the covering oxide, which for both SiO_2_ and HfO_2_ is ~900 °C. These oxides have negligible influence on the heat dissipation in either case since they are thermally insulative and extremely thin. Taken together, the difference in oxide species cannot be the cause for the 3x larger power handling capability of the CVD devices as compared to those epitaxially derived (see Fig. 1d). Furthermore, the thermal resistance—while comparable— is larger for the CVD devices than that of the epitaxial devices due to the larger interfacial thermal resistance that comes with van der Waals bonding. Thermal resistance variance is not therefore the cause for the discrepancy either. It is instead the ability of CVD devices to more uniformly generate heat thereby allowing greater power dissipation before reaching the critical degradation temperature of the surrounding oxide.

**Figure 5:**
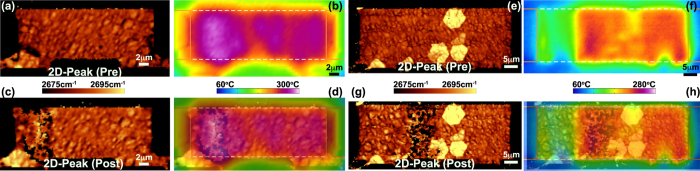
Raman and thermal imaging of two CVD covered devices failing at (**a–d**) 57 mW/*μ*m and (**e–h**) 21 mW/*μ*m (**a,e**) Raman images of 2D-mode peak position before self-heating. The 2D-mode peak position is more uniform for the higher performing device in (**a**). (**b,f**) Measured temperature distribution while dissipating ~515 mW of power. The temperature field is more uniform in (**b**) than (**f**) owing to the multilayer regions that exist in the latter. (**c,g**) Raman image of 2D-mode peak position after failure. (**d,h**) Overlay of Raman and infrared images indicating that the failure is co-located with regions of most severe heating. The grounded electrode is on the right-hand side.

This is illustrated qualitatively in the thermal maps of Fig. 5 and quantitatively in Fig. 2b. Qualitatively, Fig. 5b displays the temperature field for a CVD device where the majority of the device is heated. The more uniform heating in the CVD/Cov architecture is in contrast to the localized hot-spots that form in epitaxial devices as seen in Figs 3b and 4b. To quantify the uniformity in heating and its relationship with breakdown power, Fig. 2b plots the breakdown power versus the percentage of device area having an IR measured temperature greater than 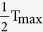. Stratification between the device architectures is evident. Specifically, the CVD/Cov devices capable of dissipating the greatest amount of power exhibit a temperature field in which the majority of the device is heated at the time of failure. In contrast, the epitaxial devices have much less heated area and are thus less capable at dissipating power.

Morphology does impact the power handling of the CVD devices as well, however. Figure 5a–d compares a higher performing CVD/Cov that failed at 57 mW/*μ*m with a device of the same architecture that failed at 21 mW/*μ*m (see Fig. 5e–h). From the thermal images in Fig. 5b,f, it is evident that the device having the larger breakdown power more uniformly heats. The Raman images of Fig. 5a,e provide an explanation for the difference. Specifically, the 2D-mode peak position varies little in Fig. 5a exhibiting only wrinkles characteristic of the transfer process^10^,^11^. For the device having lower breakdown power, Fig. 5e displays three hexagonal regions of bi-, or multilayer, graphene that evolve as part of CVD synthesis atop copper^46^. Since electrical resistance will increase at the boundaries between these and the monolayer regions, current funnels between them leading to more localized heating and thus a reduced ability to dissipate power. Thus, like that seen in the epitaxial case, CVD devices too are limited in their power handling capability based upon morphological features intrinsic to their synthesis.

At the chip scale, the power limits of a graphene device are constrained not by the thermal resistance of its surroundings but by the heat concentrating morphological features tied to its origin. Here, monolayer to bilayer transitions combined with variations in carrier concentration and mobility that arise due to the growth process have been shown to lead to localized hot spot formation that ultimately limit how much power an epitaxial graphene device can handle. While less acute, similar morphological features constrain the power handling of CVD devices as well. Thus, continued improvement in single crystal graphene synthesis^47^,^48^ will not only enhance “how well” these devices perform but also “how much” work can be performed.

## Methods

### Infrared and Raman Imaging

Infrared images were acquired utilizing a Quantum Focus Instruments system equipped with a 1024 × 1024 indium antimonide (InSb) Detector. A 20X objective having a lateral resolution of ~2 *μ*m was utilized for each measurement. Averaging over 50 frames was utilized to reduce noise. Unless otherwise noted, temperatures represent the direct output from the IR-system. This output is an average value of the probed volume and does not correspond to a temperature of any particular material. Raman spectroscopic measurements were performed with a WiTec Alpha300R Raman imaging system using a 100X/0.95NA objective resulting in beam diameters of ~350 nm when utilizing the 532 nm laser. Spectra were acquired every 225 nm and have a peak position that is accurate to within ±0.5 cm^−1^. Owing to the optical transparency and high thermal conductivity of the SiC substrate, 18 mW of power could be utilized without damaging the graphene or causing laser heating of any consequence^11^.

### Device Fabrication

Quasi-free standing epitaxial graphene monolayer was synthesized using established methodologies^29^,^49^ and fabricated into devices 5 × 15, 10 × 30, 15 × 45, and 20 × 60 *μ*m in size using standard photolithographic processes. Analogous CVD devices were fabricated by transferring ACS “Trivial Transfer” graphene onto 6H-SiC substrates using a procedure specified by the vendor. Conventional photolithographic techniques were employed to define and contact the devices^50^. For the Epi/Cov devices, 50 nm of SiO_2_ was deposited using an established plasma enhanced chemical vapor deposition process.

For the CVD/Cov devices, HfO_2_ was deposited using atomic layer deposition. To prevent graphene decomposition during the ALD process, a thin alumina layer is first grown onto the graphene surface. This protective alumina layer is formed by the controlled oxidization of a 10 Å, 0.2 Å/s electron beam evaporated aluminum thin film in pure oxygen at a partial pressure of 10 Torr. After a twenty minute exposure of the aluminum to this oxygen enriched environment, the high quality protective alumina layer is completely formed. This alumina layer not only serves to protect the graphene from potential reactants, it also appears to enhance the ALD hafnia nucleation process. Hafnia ALD was grown using a Picosun Sunale R150 hot-wall reactor operating at 250 °C using tetrakis(dimethylamido)hafnium(IV) (TDMAH) and deionized H_2_O precursors. At a source temperature of 75 °C, the TDMAH is introduced into the reaction space for a period of 1.5 s followed by a 15 s, N_2_ purge. To complete the formation of a single monolayer of hafnia, deionized water is then introduced for a period of 0.1 s followed by a 40 s, N_2_ purge. Both the TDMAH and H_2_O reaction cycles are repeated 550 times to grow a 50 nm hafnia gate dielectric.

## Additional Information

**How to cite this article**: Beechem, T. E. *et al*. Self-Heating and Failure in Scalable Graphene Devices. *Sci. Rep.* **6**, 26457; doi: 10.1038/srep26457 (2016).

## Supplementary Material

Supplementary Information
