# Supplementary material for: Self-Heating and Failure in Scalable Graphene Devices
Source: Sci Rep. 2016 Jun 9;6:26457. doi: 10.1038/srep26457 (PMC4899688; doi:10.1038/srep26457)
Supplement: Supplementary Information [file srep26457-s1.pdf]

Supplementary Information: Self-Heating and Failure in Scalable Graphene Devices

Thomas E. Beechem,* Ryan A. Shaffer, John Nogan, Taisuke Ohta, Allister B. Hamilton, Anthony E. McDonald, and Stephen W. Howell

Sandia National Laboratories, Albuquerque, New Mexico 87123, USA

E-mail: tebeech@sandia.gov

Graphene Raman Spectra

An average Raman response is provided in Figure S1 for each of the differing device architectures. The expected G- ($\sim$1580 cm^{-1}) and 2D-modes ($\sim$2680 cm^{-1}) of graphene are clearly evident as is the second order structure stemming from the underlying SiC. Largely absent, in contrast, is the defect (D) mode expected near 1350 cm^{-1}. The lack of D-peak indicates that the graphene itself is of sufficient quality and possesses comparable levels of defects irrespective of origin. Second, as these spectra were acquired after device fabrication, the small D-peak demonstrates that the processing does not appreciably damage the graphene. Differences in the power handling of the device architectures cannot, therefore, be attributed to large variations in defect level within the graphene.

*To whom correspondence should be addressed

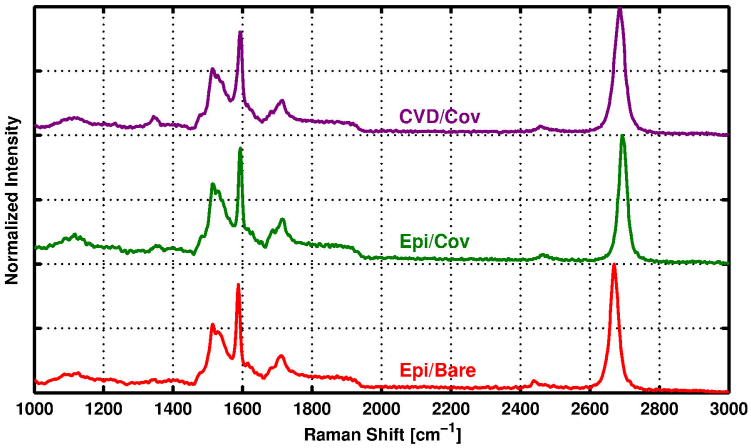

Figure S1: Average Raman spectrum from each device architecture.

Estimating Graphene Temperature from IR-Thermography

Infrared thermography is utilized to measure the temperature field of graphene devices during self-heating. To deduce temperature, the technique relates the magnitude of light emitted in the spectral band of 2-4μm to a temperature *via* the Planck distribution. Unlike other thermometry techniques that leverage material specific spectral features (*e.g.,* Raman), IR-thermography is material insensitive. It quantifies the total emittance from the device stack reaching the detector. The temperature derived from IR will therefore be an average over the volume from which signal is collected. Since most materials are at least somewhat transparent in the infrared, the collected volume will have a finite depth. Owing to this finite depth of collection, the temperature measured by IR-thermography will be less than the temperature of the atomically thin graphene. Below, a method is described to account for this volumetric averaging thereby allowing for an estimation of the graphene temperature relative to that measured by IR-thermography.

IR-thermography measures photons and deduces temperature. This deduction takes place by relating the number of photons measured from a device under test to that collected when observing a blackbody. Mathematically, this relationship can be written as:

$$\phi = \bar{\epsilon}\phi_{BB}(T_{IR}) \tag{S1}$$

where ϕ is the number photons emitted by a device during operation, $\bar{\epsilon}$ the effective emissivity of the device stack, and $\phi_{BB}(T_{IR})$ the number of photons emitted by a blackbody at the measured temperature, T_{IR}. In the following, each of these parameters is quantified to relate T_{IR} to the temperature of the graphene.

The number of photons emitted from a blackbody within a given spectral band is given by:

$$\phi_{BB} = \int_{\lambda_a}^{\lambda_b} Q(\lambda, T)\, \mathrm{d}\lambda \tag{S2}$$

where $\lambda_{b,(a)}$ represents the upper (lower) wavelength being sensed and Q is the spectral radiance given by the Planck distribution[S1] *via* :

$$Q(\lambda, T) = \frac{2\pi c}{\lambda^4} \frac{1}{\exp\left(\frac{hc}{\lambda T k_B}\right) - 1} \tag{S3}$$

where c is the speed of light, h is Planck's constant, and k_B the Boltzmann constant.

Quantifying the photons emitted from the device stack is complicated by the IR transparency of the materials involved. Specifically, the penetration depth of IR light into the oxide, graphene, and SiC is much greater than the thicknesses of the respective layers. Therefore, IR radiation generated from the entirety of the device stack is capable of reaching the detector and therefore influencing the measurement. For this reason, photons emanating from the device stack of n layers are quantified by integrating through the volume using the following relation:

$$\phi = \frac{1}{2} \sum_{j=1}^{n} \int_{z_j}^{z_{j+1}} \int_{2\mu\mathrm{m}}^{4\mu\mathrm{m}} \alpha_j e^{-\alpha_j (z - z_j)} Q(\lambda, T(z)) \gamma_j \, \mathrm{d}\lambda \mathrm{dz} \tag{S4}$$

where α_j is the absorption coefficient of the jth layer, $T(z)$ the temperature at a given depth beneath the surface, and γ the fraction of light reaching the j to $j-1$ interface at depth z_j capable of reaching the detector. $z_1 = 0$ is defined as the interface between the air and the top ($j = 1$) layer of the device stack while the pre-factor accounts for integration over only

a hemisphere.

γ_j is calculated using the transfer matrix method (TMM). Specifically, γ_j is the deduced transmission from the TMM for radiation incident from layer j that emerges from the remainder of the stack (*i.e.*, the $j-1, j-2, ...1$ layers) and into the air. The TMM is strictly valid only under the assumption of light incident in a non-absorbing medium.[S2] For SiO_2, HfO_2, and SiC, this assumption is reasonable considering the materials near transparency. The assumption breaks down in graphene, however, owing to its considerable absorption in the infrared. For this reason, TMM predictions of transmission for light originating in the graphene are non-physical (*i.e.*, $\gamma > 1$), as expected for highly absorbing films.[S3] Transmission from the graphene is therefore approximated by quantifying how much light could enter the graphene from the air. In practice, this is implemented by utilizing the TMM in an "inverted" arrangement where radiation is presumed incident from air and the amount of light transmitted into graphene is quantified. While no doubt an approximation of questionable veracity, the final results are insensitive to this parameter. For example, the curve linking the measured IR temperature to that of the graphene shown in Figure S3 changes by less than $\pm 3\%$ when transmission from the graphene is varied by $\pm 2x$. Optical constants used in the TMM calculations were obtained directly from ellipsometry measurements[S4,S5] (SiO_2, HfO_2), reference values (SiC)[S6] or, in the case of graphene, from the model of Falkovsky.[S7]

The effective emissivity of the device stack, $\bar{\epsilon}$, was quantified by utilizing Equation S4 to quantify the number of photons emanating from the stack when placed at a constant reference temperature of $T_o = 60°C$ as used in the experiment. Having the number of photons at this reference condition, ϕ_o, $\bar{\epsilon}$ is deduced *via* :

$$\bar{\epsilon} = \frac{\phi_o}{\phi_{BB}(T_o)} \tag{S5}$$

Using Equations S1 - S5, the temperature of the graphene can be linked to that measured by the IR-thermography measurement provided that the temperature gradient, $T(z)$, through

the thickness of the device stack is known. To this end, Joule-heating of the graphene device was simulated using ANSYS-based finite element analysis. Specifically, three-dimensional temperature fields for the Epi-Bare device geometry shown in Figure 1 of the main text were predicted under the following methodology.

First, temperature dependent thermal conductivities were employed for each of the layers, namely graphene,[S8] metal contact[S9] and SiC.[S10] Kapitza conductance between the metal contact and graphene was assumed to be 25 MW/m^{2}K in line with Hopkins et al.[S11] and 85 MW/m^{2}K between the graphene and SiC.[S12] All boundaries were defined as adiabatic except for the bottom SiC surface that was specified to remain at the reference temperature of $60°C$. The size of the domain and mesh were varied until temperatures changed by less than 1%.

Uniform heat generation was assumed within the graphene. As discussed in the text, uniform heat generation does not occur in actuality owing to graphene's imperfect morphology. Here, however, the aim is not to predict the exact temperature field for a particular device possessing a particular morphology. Instead, quantification of the the through thickness temperature (*i.e.*, T(z)) at the location of maximum heating is the goal as this provides the necessary information to link the maximum measured IR temperature to that of the graphene. Finally, the oxide was not included in the simulation as—owing to its thinness and low thermal conductivity—it is of negligible consequence to heat dissipation. Devices possessing an oxide overlayer will thus have nearly identical temperature profiles as those exposed to the atmosphere.

From these simulations, Figure S2(a) provides the through thickness temperature field at the mid-point of a graphene device. Temperature decays rapidly from beneath the graphene reaching half the maximum value at a depth only 8 μm below the surface (See Figure S2(b)). IR-thermography samples much deeper than this heated region, however, as transmission through the entire device stack is $> 75\%$ as calculated via the TMM. Thus, there will be a non-negligible number of photons from the deeper, cooler, region of the device that reach the

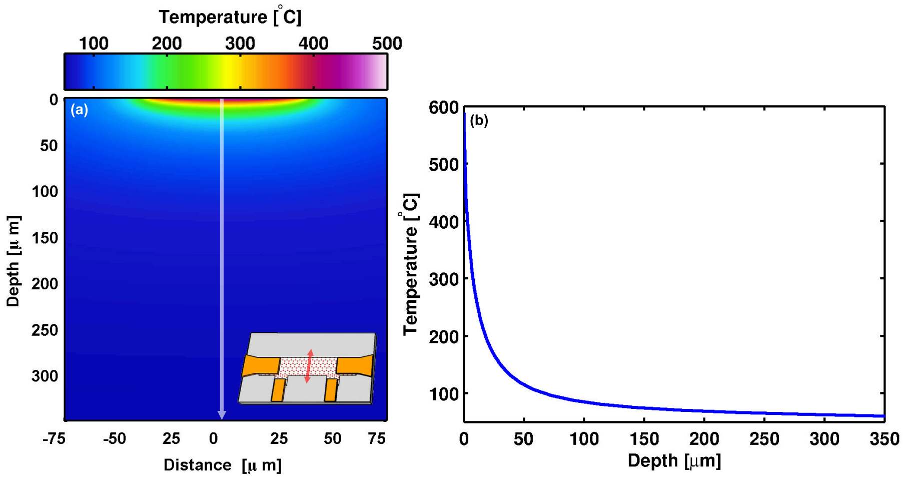

Figure S2: (a) Simulated through thickness temperature distribution along the center-line of a graphene device dissipating 600 mW. Inset: Red arrow indicates location of through-thickness plane. (b) Temperature profile through the thickness of the device along the white arrow of (a). Owing to the rapid temperature decay and the sampling of the IR measurement from the entirety of the device stack, the measured IR temperature is much less than the temperature of the graphene.

detector. The resulting temperature measurement will then be some volumetrically averaged value having a magnitude between the maximum surface temperature (*i.e.*, the graphene) and the reference value.

To quantify the extent of volumetric averaging, the through thickness temperature profile shown in Figure S2(b) is input into Equation S4 as T(z) to quantify the number of photons (ϕ) reaching the detector during heating. With both the number of photons and the effective emissivity ($\bar{\epsilon}$) previously calculated, Equation S1 can be solved to obtain an estimate of the IR-measured temperature (T_{IR}). Repeating this process for the range of powers explored experimentally, Figure S3 relates the temperature deduced from IR-thermography to that of graphene. Similar to the deductions made by Bae et al.,[S13] graphene's temperature is much greater than that obtained from the measurement. Quantitatively, graphene's temperature is $\sim 3x$ greater than that deduced *via* IR-thermography. Using the curve of Figure S3, the maximum temperature at failure measured by IR-thermography shown in Figure 2(a) of the main text is used as an estimate of the temperature of graphene at failure. The resulting average values for each device type are subsequently displayed as the inset to Figure 2(a).

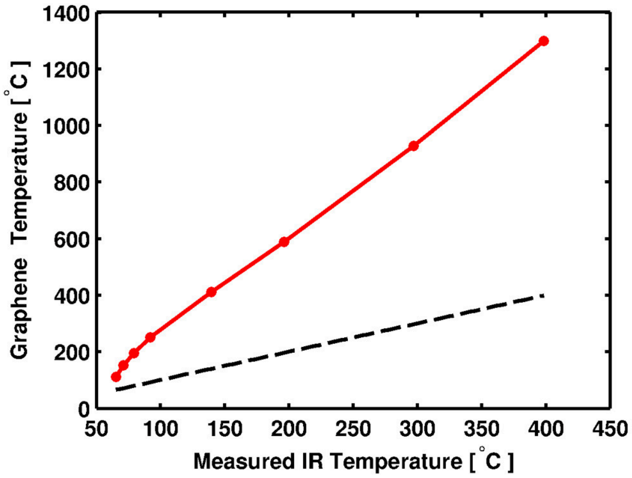

Figure S3: Estimated temperature of graphene versus that deduced from IR-thermography. Owing to volumetric averaging, the graphene is at a temperature $\sim 3x$ greater than the measured temperature. The dashed curve is the line of equality.

References

[S1] Chen, G. *Nanoscale Energy Transport and Conversion*; Oxford University Press: New York, NY, 2005.

[S2] Centurioni, E. *Appl. Opt.* **2005**, *44*, 7532–7539.

[S3] Knittl, Z. *Optics of Thin Films: an Optical Multilayer Theory*; Wiley London:, 1976.

[S4] Shelton, D.; Peters, D.; Sinclair, M.; Brener, I.; Warne, L.; Basilio, L.; Coffey, K.; Boreman, G. *Opt. Express* **2010**, *18*, 1085–1090.

[S5] Ginn, J. C.; Jarecki Jr, R. L.; Shaner, E. A.; Davids, P. S. *J. Appl. Phys.* **2011**, *110*, 043110.

[S6] Palik, E. D. *Handbook of Optical Constants of Solids*; Academic press, 1998; Vol. 3.

[S7] Falkovsky, L. *J. Phys.: Conf. Ser.* **2008**, *129*, 012004.

[S8] Seol, J.; Jo, I.; Moore, A.; Lindsay, L.; Aitken, Z.; Pettes, M.; Li, X.; Yao, Z.; Huang, R.; Broido, D. *Science* **2010**, *328*, 213.

[S9] CINDAS, Thermophysical Properties of Matter Database. 2014.

[S10] Su, G.-P.; Zheng, X.-H.; Qiu, L.; Tang, D.-W.; Zhu, J. *Int. J. Thermophys.* **2013**, *34*, 2334–2342.

[S11] Hopkins, P. E.; Baraket, M.; Barnat, E. V.; Beechem, T. E.; Kearney, S. P.; Duda, J. C.; Robinson, J. T.; Walton, S. G. *Nano Lett.* **2012**, *12*, 590–595.

[S12] Xu, Z.; Buehler, M. J. *J. Phys.: Condens. Matter* **2012**, *24*, 475305.

[S13] Bae, M.-H.; Ong, Z.-Y.; Estrada, D.; Pop, E. *Nano Lett.* **2010**, *10*, 4787–4793.
